# Remote Video Assessment of Ataxia via SARA^home^ : Correlation of Pose‐Derived Endpoints with Clinician Severity Ratings

**DOI:** 10.1002/mdc3.70854

**Published:** 2026-10-16

**Authors:** Rabia Aziza, Elisa Ferrer, Fereshteh Poursaeed, Vivian Maas, Jennifer Faber, Arian Taheri Amin, Katharina Hill, Matthis Synofzik, Lisa H. Graf, Dagmar Timmann, Andreas Thieme, Paola Giunti, Hector Garcia‐Moreno, Magda M. Santana, Jeannette Hübener‐Schmid, Elin H. Davies, Thomas Klockgether, Marcus Grobe‐Einsler

**Affiliations:** ^1^ Aparito—a wholly owned subsidiary of Eli Lilly and Company Wrexham United Kingdom; ^2^ German Center for Neurodegenerative Diseases (DZNE) Bonn Germany; ^3^ Department of Parkinson's Disease, Sleep and Movement Disorders University Hospital Bonn Bonn Germany; ^4^ Department of Neuroradiology University Hospital Bonn Bonn Germany; ^5^ Center for Neurology and Hertie Institute for Clinical Brain Research University of Tübingen Tübingen Germany; ^6^ German Center of Neurodegenerative Diseases (DZNE) Tübingen Germany; ^7^ Department of Neurology and Center for Translational Neuro‐ and Behavioral Sciences (C‐TNBS) Essen University Hospital, University of Duisburg‐Essen Essen Germany; ^8^ Ataxia Centre, Department of Clinical and Movement Neurosciences, UCL Queen Square Institute of Neurology University College London London United Kingdom; ^9^ Center for Neuroscience and Cell Biology University of Coimbra (CNC‐UC) Coimbra Portugal; ^10^ Center for Innovative in Biomedicine and Biotechnology (CIBB) University of Coimbra Coimbra Portugal; ^11^ Gene Therapy Center of Excellence (GeneT) Coimbra Portugal; ^12^ Institute for Medical Genetics and Applied Genomics University of Tübingen Tübingen Germany; ^13^ Department of Neurology, Center for Movement Disorders and Neuromodulation, Medical Faculty Heinrich‐Heine University Düsseldorf Germany

**Keywords:** ataxia, digital biomarkers, gait analysis, pose estimation, remote assessment, SARA^home^

## Abstract

**Background:**

Remote assessment of ataxia is increasingly important for patient‐centered care and clinical research. SARA^home^, a validated video‐based adaptation of the Scale for the Assessment and Rating of Ataxia (SARA), enables self‐administration of key motor tasks at home.

**Objective:**

The aim was to evaluate the correlation of pose‐derived gait and stance endpoints from SARA^home^ videos with clinician‐rated severity.

**Methods:**

In a multisite observational study, participants recorded SARA^home^ tasks on consumer mobile devices via the Atom5^TM^ app. Clinicians rated videos using standardized criteria, blinded to automated outputs. Kinematic measures were extracted via pose estimation and aggregated to video‐level features. Videos were aggregated per participant before correlation using Spearman's *ρ* and Kendall's *τ*, with 95% cluster‐bootstrap confidence intervals.

**Results:**

We analyzed 5418 gait and stance videos from 58 participants across 4 sites (gait n = 2205, stance n = 3213). Score distribution was skewed toward lower severity. For gait, clearance variability (*ρ* = 0.52 [0.37–0.64]) and median‐normalized base of support (*ρ* = 0.51 [0.35–0.66]) correlated most strongly with item ratings, followed by shoulder sway root mean square (*ρ* = 0.43 [0.24–0.60]). Adjusted foot clearance (worse limb) correlated negatively (*ρ* = −0.35 [−0.52 to −0.15]). For stance, anteroposterior sway (*ρ* = 0.51 [0.36–0.65]), leg alignment variability (*ρ* = 0.51 [0.36–0.65]), and composite sway (*ρ* = 0.48 [0.32–0.62]) were most associated with severity. Base of support widened monotonically with severity and was the most reliable gait feature (intraclass correlation coefficient [ICC] = 0.88).

**Conclusions:**

Pose‐derived digital endpoints from home‐captured SARA^home^ videos show robust, clinically interpretable correlation with severity, supporting scalable remote phenotyping. Findings motivate longitudinal validation toward clinical and regulatory utility.

Ataxias are rare neurodegenerative movement disorders characterized by impaired balance, coordination, and speech. Limited mobility, defined by impaired gait and stance, ranges among the most burdensome symptoms presented by ataxia patients and correlates with overall disease severity.^1^, ^2^ Impairments result in reduced participation in social activities and health‐related quality of life.^3^, ^4^, ^5^, ^6^ With a few exceptions, there are no treatments able to stop or delay disease progression,^7^ but the recent approval of omaveloxolone^8^ for Friedreich's ataxia represents a milestone for drug development in this field. Spinocerebellar ataxia type 3 (SCA3), which is caused by CAG repeat mutations in the *ATXN3* gene, is one of the most common forms of dominantly inherited ataxia worldwide.^9^ Targeted therapeutic approaches, such as antisense oligonucleotides, are currently being developed and tested in clinical trials. However, long journeys and motor impairments are significant restrictions to the participation of ataxia patients in clinical trials. Therefore, remote objective evaluation is increasingly important in these patients, as it reduces travel burden and supports patient‐centered care. SARA^home^, a validated video‐based adaptation of the Scale for the Assessment and Rating of Ataxia (SARA),^1^ enables patients to self‐administer 5 core items—gait, stance, speech, nose–finger test, and fast alternating hand movements—at home.^10^ Using video‐based remote assessments, such as SARA^home^, in longitudinal, multicenter studies produces substantial quantities of image data. Recent advances in computer vision (ie, automated image and video analysis) and machine learning have enabled automated movement analysis from clinical videos, supporting objective quantification of motor impairment.^11^, ^12^ Pose‐estimation approaches have already shown promise in ataxia: video‐based kinematic analysis detected drug‐related changes in movement quality in a phase 3 spinocerebellar ataxia trial,^22^ and a MoveNet‐based pipeline with regression models estimated ataxia severity from gait videos.^23^ However, these efforts relied on in‐clinic or supervised recordings; the feasibility of fully home‐recorded, unsupervised capture and the automated curation required to make such heterogeneous data analyzable remain largely unaddressed.

Although automated video analysis offers a promising avenue for cost‐ and time‐efficient remote assessments in clinical trials, its applicability to home‐recorded data remains uncertain due to environmental variability. This feasibility study evaluates whether pose‐estimation‐based (ie, automated body‐joint tracking) analyses of gait and stance recordings of SARA^home^ can yield robust, interpretable digital measures despite such challenges and derive potential digital endpoints. We evaluate whether these automated measures correlate with disease severity and score‐level trends consistent with expert ratings, aiming to establish reliable, scalable digital biomarkers for remote ataxia monitoring.^10^, ^11^

## Patients and Methods

### Study Design and Setting

This analysis was an academic–industry collaboration between Aparito and the German Center for Neurodegenerative Diseases (DZNE). Patients were recruited from study centers of the European Spinocerebellar Ataxia Type 3/Machado‐Joseph‐Disease Initiative (ESMI) in Germany (Bonn, Essen, Tübingen) and the United Kingdom (London). The study was approved by the local ethics committees of all participating sites and conducted in accordance with the Declaration of Helsinki. All patients provided written informed consent prior to participation.

Participants completed SARA^home^ tasks twice daily for 2 weeks at home with loaner consumer mobile devices (iPads)^13^; videos were uploaded to the Atom5^TM^ platform for remote clinical review and scoring, followed by automated pose‐estimation‐based analysis. Trained raters scoring the videos were blinded to automated outputs.

SARA^home^ operationalizes 5 SARA items feasible in the home setting: gait, stance, speech, nose–finger, and fast alternating hand movements.^10^ The current analysis centers on gait (normal and tandem) and stance (feet apart or normal, feet together, and tandem). The full pipeline is summarized in Figure 1, and task illustrations are provided in Figure S1.

**Fig 1:**
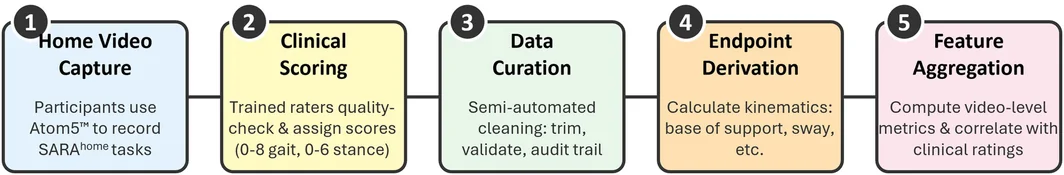
End‐to‐end pipeline. Step 1: participants record SARA^home^ tasks at home using the Atom5^TM^ app on loaner iPads. Step 2: clinician raters review and score videos via the Atom5^TM^ portal. Step 3: semiautomated data curation ensures quality. Step 4: pose estimation extracts body‐joint landmarks. Step 5: digital endpoints and video‐level features are derived and correlated with clinical ratings.

### Participants and Capture

Fifty‐eight SCA3 mutation carriers (30 women; mean age: 50.4 [standard deviation, SD, 11.1] years, range: 26–79) were recruited from 4 study centers in Germany (Bonn, Essen, Tübingen) and the United Kingdom (London). Baseline scores on the standard clinician‐administered SARA (total range: 0–40) ranged from 0 to 25.5 (mean 9.3 [SD 6.1]); these whole‐scale scores characterize the cohort and are distinct from the per‐item SARA^home^ scores used as the reference for the digital endpoints. The cohort included both baseline assessments and 1‐year follow‐up visits for a subset of participants, primarily from the Bonn site, as described in the companion SARA^home^ study.^13^ In the present analysis, all recordings from a participant were aggregated to the median per participant and score level before correlation, so that repeated videos were not treated as independent (see Patients and Methods). Participant demographics are summarized in Table 1.

**TABLE 1:** Cohort characteristics

Characteristic	Overall (N = 58)
Age, mean (SD), years	50.4 (11.1)
Age, range	26–79
Sex, female, n (%)	30 (53.6%)
SARA total score (standard scale, 0–40), mean (SD)	9.3 (6.1)
SARA total score (standard scale, 0–40), median (range)	9.0 (0–25.5)
Site	
Bonn, n	20
London, n	20
Tübingen, n	10
Essen, n	6

Abbreviations: SARA, Scale for the Assessment and Rating of Ataxia; SD, standard deviation.

Within Atom5^TM^, participants received standardized instructions (eg, camera distance and angle, stable support surface, avoidance of backlighting). Recordings were performed using loaner tablets (iPads) and securely uploaded to Atom5^TM^ servers. The capture workflow emphasized the participant's full‐body visibility for kinematic analysis and safety (eg, presence of a caregiver if needed).

### Clinical Assessment and Scoring

Trained raters accessed the Atom5^TM^ web portal (see Fig. S2) to perform quality checks and assign SARA^home^ item scores to each video according to standardized criteria: gait tasks were scored on a 0 to 8 scale, with 0 indicating normal and 8 unable to walk even with support, and stance was scored on a 0 to 6 scale, with 0 indicating normal tandem >10 seconds and 6 unable to stand >10 seconds even with support.

### Data Curation

A semiautomated preprocessing workflow was applied prior to automated analysis to ensure data integrity: removal of nontask segments, verification of the correct individual in multiperson frames (Fig. S3), and exclusion of videos not meeting minimum task duration or visibility requirements. All edits and exclusions were logged to maintain an auditable trail. (details about the data cleaning process are presented in Appendix S1).

### Outcomes and Digital Endpoints

SARA^home^ protocol incorporates measurable digital endpoints from gait and stance tasks for objective, remote assessment of ataxia severity.

The automated analysis pipeline involves a 3‐step process to derive clinically relevant metrics:

- Landmarks: OpenPose,^21^ a pose‐estimation framework powered by deep learning models, was used to track body landmarks, generating a set of coordinate time‐series values across video frames. OpenPose was selected for its validated accuracy in multiperson two‐dimensional landmark estimation and its established use in clinical gait and movement‐disorder video analysis; the pipeline is, however, pose estimator agnostic, and other frameworks (eg, MoveNet, BlazePose) can be substituted without altering the downstream feature extraction.
- Digital endpoints: these landmarks are used to calculate digital endpoints (eg, base of support, trunk sway angle), which are time‐series values representing core physical measures throughout the task.
- Video‐level features: finally, these time‐series endpoints are aggregated to calculate video‐level features (eg, median, variability, root mean square [RMS]), which are single‐value metrics used to quantify characteristics such as the amplitude, smoothness, or symmetry of the movement over the entire video.

In this paper, we focus on kinematic endpoints—geometric measures that capture distance and angular relationships between key joints—providing interpretable features, rather than temporal/event‐related endpoints such as duration of walking or speed.

The processing pipeline of endpoints included the following:

- Gap filling: missing values were interpolated using time‐weighted methods.
- Smoothing: a moving average filter was applied to reduce frame‐to‐frame noise.
- Outlier removal: outliers were detected using Isolation Forest and replaced with interpolated values.
- Normalization: distance‐based measures were normalized by shoulder width to account for body size and camera perspective.
- Turn detection: for normal gait tasks, turns were identified using a combination of principal component analysis (PCA)–based arm swing analysis and normalized foot elevation signals from both limbs. The combined elevation series was smoothed using a Savitzky–Golay filter, and peaks were detected based on prominence relative to signal variability; the largest peak marked the turn center. Start and end frames were determined using a dynamic threshold derived from baseline variability, filling short gaps to ensure continuity. Turn segments were excluded from feature aggregation.

Gap filling, smoothing, and outlier removal were applied uniformly across all videos irrespective of clinical scores. Outlier detection used Isolation Forest fitted independently for each video and each measure on its individual smoothed signal (univariate), with no train/test split and no global or cross‐video training; flagged frames were set to missing and re‐interpolated. The contamination parameter (the assumed proportion of anomalous frames) was fixed at 0.05, reflecting the low rate of gross tracking errors expected after quality control and smoothing, and the random seed was held constant for reproducibility. Because the model is fitted and applied within each individual time series, no learned representation can transfer dataset or site‐specific bias across recordings; and because the algorithm is unsupervised and interpolation is locally constrained, these steps mitigate pose‐estimation artifacts without introducing score‐dependent bias.

The selected endpoints reflect core domains of motor function, encompassing balance/coordination (trunk sway, shoulder sway, arm movement) and lower‐limb function (base of support, foot elevation, and leg alignment through knee flexion). Several of these endpoints have established clinical correlates in ataxia assessments and are therefore expected to provide interpretable indicators of postural control, suitable for systematic evaluation of disease severity in a home setting.

Finally, we aggregated the frame‐wise kinematic endpoints into clinically interpretable video‐level features. Aggregation included measures of central tendency (eg, medians), variability metrics (eg interquartile range), and symmetry indices where appropriate. Gait and stance tasks were aggregated separately; for gait, turns were excluded using PCA‐based and foot‐elevation‐based detection to avoid confounding. These features were designed to capture task‐specific movement characteristics while accounting for artifacts such as turns.

To evaluate the association of each feature with clinician‐rated severity, Spearman's *ρ* and Kendall's *τ* were calculated between each feature and the SARA^home^ item scores, separately for gait and stance tasks. Because each participant contributed multiple recordings, features were first aggregated to the median value per participant and score level, so that observations were not treated as independent across repeated videos; correlations were computed on these participant‐level values, and 95% confidence intervals (CI) were obtained by cluster‐bootstrap resampling over participants (2000 iterations). As a sensitivity analysis, correlations were recomputed using a single median value per participant, which yielded consistent estimates (Table S1). For each feature we report the a priori most informative subtask (normal walking for gait, tandem stance for stance); full per‐subtask results are provided in Table S1.

Test–retest reliability of each video‐level feature was quantified using the intraclass correlation coefficient (ICC) (1); one‐way random effects, single measurement, and absolute agreement were computed separately for each feature and subtask. Repeated recordings collected within the baseline window (within 30 days of each participant's first recording), over which disease severity is assumed stable, were treated as replicates; 95% CIs were obtained by cluster‐bootstrap resampling of participants (2000 iterations), and reliability was interpreted using established thresholds^24^ (poor: <0.50, moderate: 0.50–0.75, good: 0.75–0.90, excellent: >0.90). As a sensitivity analysis, ICCs were reestimated on participant–subtask cells with a constant clinician score. Full results are provided in Table S2. Details of the calculations are provided in Appendix S2.

## Results

### Dataset and Score Distribution

Each participant performed a range of gait and stance tasks, resulting in 2205 gait videos (1107 normal walking, 1098 tandem walking) and 3213 stance videos (1080 normal stance, 1065 feet together, 1068 tandem stance).

Disease severity was assessed using clinician‐rated SARA^home^ item scores. The distribution of scores is skewed toward lower severity levels, with fewer videos available for higher scores (see Fig. S4). This reflects the increasing difficulty with task completion as disease progresses.

### Digital Endpoint Derivation

For each video, pose‐estimation outputs were processed to derive digital endpoints corresponding to core SARA^home^ assessment items. Higher SARA^home^ gait scores are associated with wider ankle separation, reflecting compensatory strategies for balance, as illustrated by the progression of base of support across severity levels (see Fig. S5). However, it is notable that at gait item score 7, the foot distance is smaller than at score 5, likely due to increased instability or reliance on assistive devices at more severe stages.

An example of the stance task is provided in Figure S6.

Accurate segmentation of turns is essential to prevent inflated variability in straight‐walking performance, as turns introduce variability unrelated to gait stability. An example of the resulting turn segmentation for a single subject with severe gait impairment (SARA^home^ gait item score = 5) is shown in Figure S7. This illustrates how the turn‐detection step isolates and removes the turn interval so that downstream variability metrics (eg, clearance variability, leg alignment variability) reflect straight‐walking performance rather than turn‐related motion.

In severe cases (eg, score 5), angular measures such as knee flexion and trunk sway show pronounced fluctuations compared to mild cases, suggesting increased reliance on trunk and limb adjustments for stability.

Figure 2 shows distributions of the median‐normalized base‐of‐support measure across SARA^home^ item score levels, confirming systematic shifts with disease severity. Median base of support generally increases with severity, underscoring its potential as a primary digital biomarker for disease progression.

**Fig 2:**
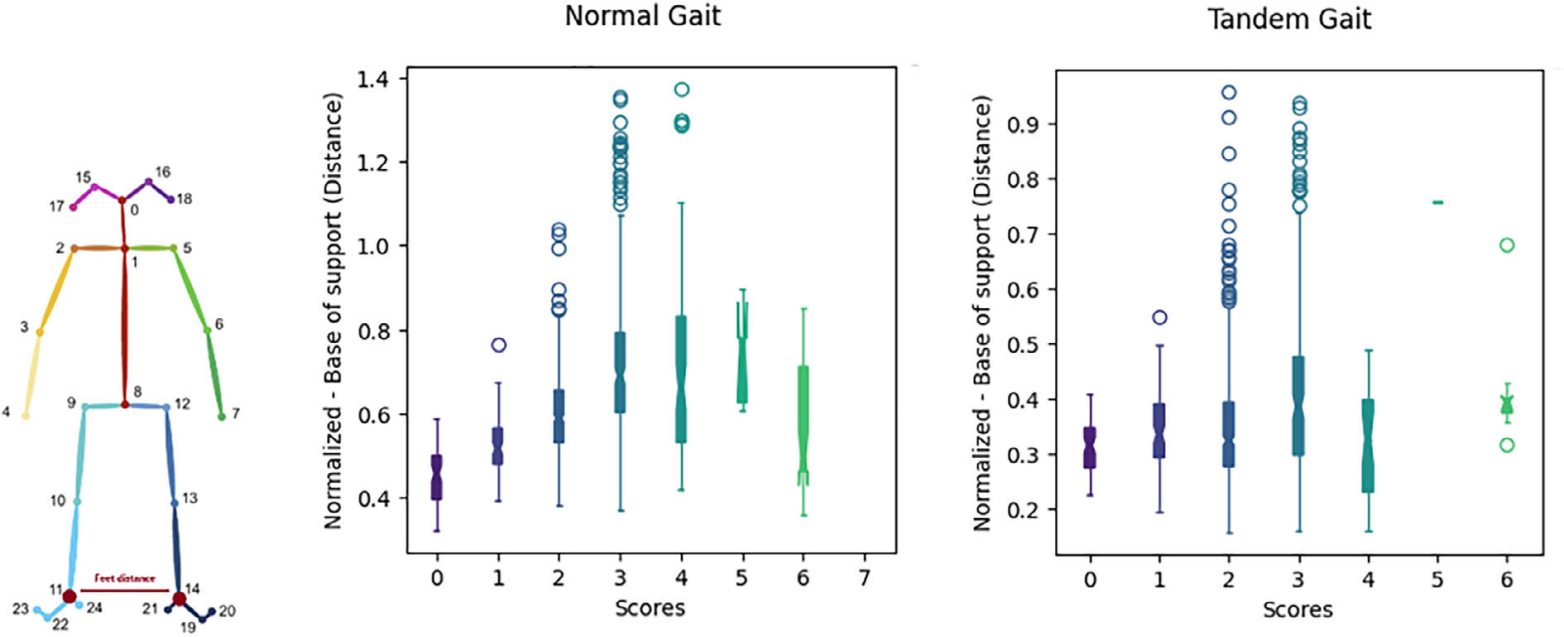
Distribution of the median‐normalized base of support across SARA^home^ gait item scores for normal gait (left) and tandem gait (right). Each box plot represents all videos at a given score level. Higher scores correspond to greater severity.

### Feature Behavior Across SARA^home^ Item Scores

We derived video‐level features from cleaned and normalized measures, grouped by gait and stance tasks. Here, we detail the feature set and summarize their behavior across severity levels.

Not all features exhibited monotonic trends with severity, reflecting the heterogeneity of compensatory strategies and the limited sample at higher score levels.

For gait, the feature set comprised adjusted clearance of the worse limb (ie, the limb with the lower median hip‐to‐ankle distance, normalized by shoulder width), clearance asymmetry, clearance variability, arm swing amplitude, arm swing asymmetry, shoulder sway RMS, median‐normalized base of support, and leg alignment variability; their trends across SARA^home^ gait item scores are shown in Figure 3. For stance, features included mediolateral sway RMS, anteroposterior sway RMS, composite sway, mediolateral sway velocity, median‐normalized base of support, and leg alignment variability; their shifts across SARA^home^ stance item scores are shown in Figure S8.

**Fig 3:**
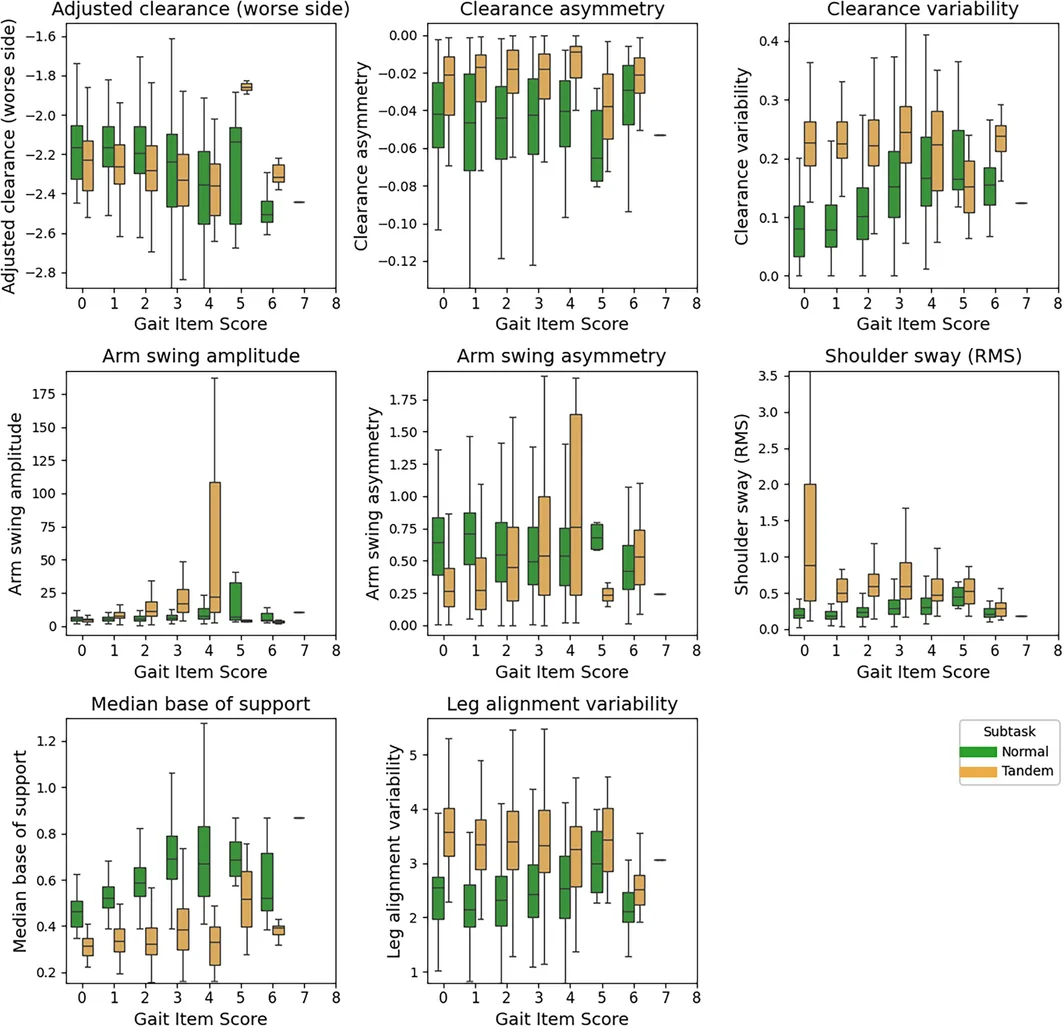
Features aggregated per SARA^home^ item score for gait tasks (normal walking excluding turns and tandem walking).

Feature ranking analysis (Fig. 4) revealed systematic associations between digital features and disease severity. For gait (normal walking), clearance variability (Spearman's *ρ* = 0.52, 95% CI 0.37–0.64) and median‐normalized base of support (*ρ* = 0.51, 95% CI 0.35–0.66) exhibited relatively stronger positive correlations with SARA^home^ item score, followed by shoulder sway RMS (*ρ* = 0.43, 95% CI 0.24–0.60). Adjusted clearance of the worse limb exhibited a moderate negative correlation (*ρ* = −0.35, 95% CI −0.52 to −0.15), indicating reduced foot clearance with increasing severity. Arm swing amplitude also exhibited significant but weaker associations (*ρ* = 0.34, 95% CI 0.14–0.52).

**Fig 4:**
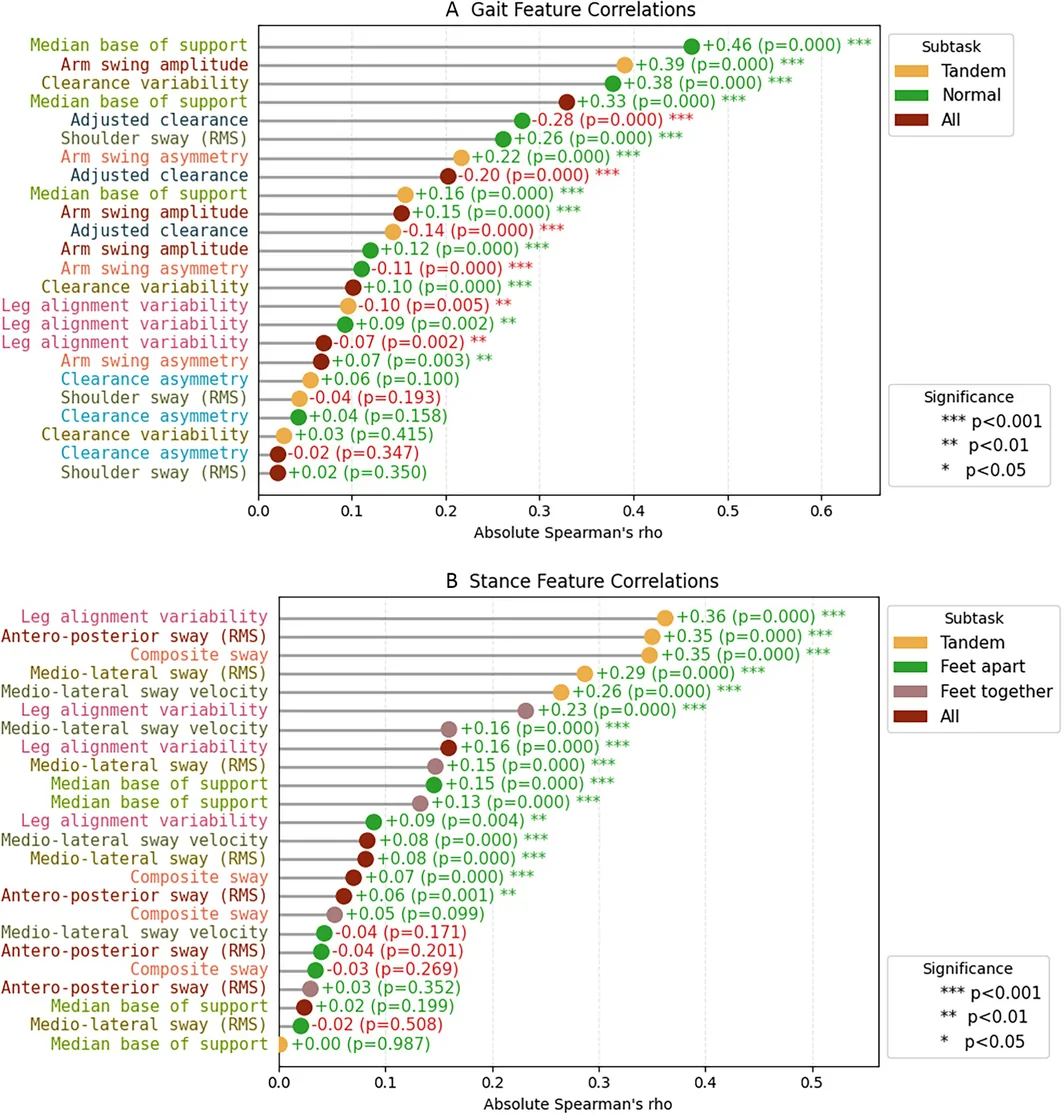
Participant‐level Spearman's ρ (with 95% cluster‐bootstrap confidence intervals) between video‐level features and clinician‐rated scores for **(A)** gait and **(B)** stance tasks. Correlations were computed after aggregating repeated recordings to the median per participant and score level. Colors denote subtask: normal (green), tandem (orange), feet apart (warm rose), and combined (dark red).

For stance (tandem task), the strongest correlations were observed for anteroposterior sway RMS (*ρ* = 0.51, 95% CI 0.36–0.65), leg alignment variability (*ρ* = 0.51, 95% CI 0.36–0.65), and composite sway (*ρ* = 0.48, 95% CI 0.32–0.62). Mediolateral sway RMS also exhibited positive association (*ρ* = 0.26, 95% CI 0.05–0.45). Test–retest reliability varied by feature and did not simply track correlation strength. Median‐normalized base of support in normal gait exhibited good reliability (ICC = 0.88, 95% CI 0.72–0.93), and adjusted clearance of the worse limb was moderately reliable (ICC = 0.74, 95% CI 0.63–0.80); base of support remained moderately reliable in tandem gait (ICC = 0.71) and in the feet‐apart and tandem stance conditions (ICC ≈ 0.53). By contrast, the variability and sway‐based features (including clearance variability and leg alignment variability, despite their comparatively strong correlations) exhibited poor single‐recording reliability (ICC <0.50), consistent with their greater frame‐to‐frame noise and underscoring the need to aggregate repeated recordings per participant. Full per‐subtask reliability estimates are shown in Figure S9 and Table S2.

## Discussion

Our findings demonstrate that pose‐derived digital endpoints from home‐captured SARA^home^ videos correlate with clinician‐rated ataxia severity and show systematic score‐level trends.

### Comparison with Traditional Methods

This study demonstrates that automated video analysis can generate metrics that correlate with established clinician ratings from real‐world home recordings of gait and stance tests to complement standardized clinical assessments in patients with SCA3. By leveraging pose estimation and machine learning, this approach extends digital phenotyping to motor domains and aligns with broader trends in remote neurological assessment. Unlike traditional in‐person SARA ratings, which require trained clinicians and scheduled clinic visits, automated extraction from home videos enables scalable, frequent monitoring without additional rater burden, a critical advantage when longitudinal studies generate large numbers of assessment videos.^11^, ^14^, ^15^ No single feature is intended to replace the clinical review of the recording; each is a simple, interpretable measure suited to frequent, repeated home tracking. The principal contribution of this work is the automated curation and preprocessing pipeline—multiperson disambiguation, unsupervised outlier handling, turn detection, and body‐size normalization—that renders heterogeneous home‐recorded video analyzable at scale; the observed correlations with clinician ratings serve as proof of principle that this pipeline preserves clinically meaningful signal.

### Feasibility

Remote home assessments are subject to greater variability in terms of the quality of performance and recording, which interferes with the automation of evaluation. We established a robust preprocessing pipeline, including interpolation, smoothing, outlier removal, and normalization, to ensure data quality. The significant correlations between pose‐derived features and clinician ratings demonstrate that algorithmic handling of noisy home‐recorded video data can yield consistent feature aggregation across participants and settings. Despite variability in lighting, camera angles, and backgrounds, preprocessing steps (interpolation, smoothing, outlier removal, normalization) yielded features with systematic score‐level trends and significant correlations with clinical ratings (eg, median‐normalized base of support *ρ* = 0.51, 95% CI 0.35–0.66), indicating robust extraction of clinically meaningful signals. We demonstrate that recognition of complex kinematic events such as turns during gait can be recognized and isolated without manual intervention. Based on the preprocessed data, we derived meaningful digital measures from pose estimation that correlate with SARA^home^ item‐level scores. Moreover, longitudinal home assessment produces large numbers of videos, and manual ratings are time intensive and expensive in the context of clinical trials.

These effects persisted despite heterogeneous home environments, indicating that carefully curated video data can support scalable, remote phenotyping.

### Gait Adaptations and Clinical Implications

Widening of base of support with increasing SARA^home^ item scores (Fig. 2) reflects compensatory strategies for stability in ataxia.^10^, ^12^, ^14^ These patterns reinforce the clinical relevance of base of support and limb‐displacement amplitude. Movements in severe cases appear irregular and erratic, with greater amplitude of limb displacement as the disease progresses; however, this amplitude tends to decline when assistive devices are used.

These kinematic patterns, captured by pose‐derived endpoints, support remote monitoring and patient care (eg, assessing the need for assistive devices or preventing falls), suggesting value for longitudinal tracking, cohort stratification, and sensitivity analyses in studies where frequent clinic visits are impractical.^14^, ^15^, ^16^

### Digital Feature Trends and Ranking

Progressive changes in digital features across SARA^home^ item scores (Figs. 2 and Fig 3) confirm their association with disease severity. Base of support emerged as the most robust gait indicator, pairing a strong severity correlation with good test–retest reliability. Leg alignment variability was the leading stance correlate but, like the other variability‐ and sway‐based features, exhibited lower single‐recording reliability, so stance endpoints appear less mature and depend more on aggregation of repeated recordings. Both associations are nonetheless in line with previous studies.^2^, ^14^, ^17^, ^18^ Feature ranking analyses (Fig. 4) reinforce the clinical relevance and face validity of these biomarkers for remote assessment.^12^ These results highlight the capacity of pose‐derived digital endpoints to capture clinically meaningful trends across SARA^home^ item score strata.

## Limitations

The dataset is skewed toward lower severity, and pose‐estimation errors may affect measurement accuracy, especially in challenging video conditions. The cohort is modest (58 participants), and videos at higher‐severity levels are sparse (recordings at the most severe gait scores represent only a small fraction of the dataset, ~2.8%), so findings at higher score levels should be regarded as exploratory and hypothesis generating.

In addition, correlations were estimated after aggregating repeated recordings to the participant level to respect the nonindependence of multiple videos per participant. A preliminary within‐subject analysis of the participants with a repeat visit is reported here; formal longitudinal modeling of responsiveness to change remains a priority for future work. Test–retest reliability was feature dependent: the leading gait biomarkers were moderate to well reliable, whereas variability‐ and sway‐based features exhibited lower single‐recording reliability (Table S2), reinforcing the value of aggregating repeated recordings.

Although preprocessing mitigates some issues, further refinement of algorithms and protocols is needed. Video‐based assessments may be less feasible for patients with advanced mobility impairment or cognitive impairment, underscoring the need for adaptive technologies and inclusive protocols.^12^

To probe whether within‐participant trajectories track clinician scores, we examined the subset of participants with a repeat visit. Eight participants (all from the Bonn site) contributed a second assessment ~1 year after baseline. A few participants changed their SARA^home^ item score between visits (2–3 per subtask), and every such change reflected worsening. Among these, change in the corresponding leading feature was concordant with the score change in 5 of the 11 subtask‐level changes and was mixed overall (Fig. S10; Table S3). For normal gait, the participant with the largest increase in score (+3) exhibited a narrowing rather than widening of base of support, consistent with this participant's use of walking support at the follow‐up visit (recorded in the rating metadata) that was not required at baseline, which reduces gait excursion and narrows the base of support despite worsening ataxia. These observations are exploratory and constrained by the small number of participants whose score changed, a single contributing site, and the lower single‐recording reliability of the variability‐ and sway‐based features (ICC <0.50; Table S2). Formal characterization of responsiveness to change will require a larger, multisite longitudinal sample.

## Future Directions

Expanding the dataset to include higher‐severity participants and validating endpoints across diverse cohorts will strengthen generalizability. Although this study demonstrates feasibility for gait and stance automation, extending the approach to remaining SARA^home^ items (speech, nose–finger test, fast alternating hand movements) is needed to enable full composite score automation and would also capture additional ataxia features beyond gait and balance—including dysdiadochokinesis, dysmetria, and tremor—that significantly affect daily function. Beyond single‐feature associations, combining the pose‐derived features in a multivariate model (eg, ordinal or ensemble regression with participant‐level cross‐validation) may estimate overall severity more directly than any individual endpoint; we view this as a natural next step once larger, more severity‐balanced samples are available. Multimodal validation combining video analytics with wearable sensor data would both validate pose‐derived measures against sensor‐based kinematic ground truth and enhance measurement precision for subtle kinematic features.^10^, ^13^ Standardized training resources for video capture^19^, ^20^ and normative reference datasets will be essential for regulatory qualification and clinical adoption of video‐derived digital endpoints.

Taken together, these results position SARA^home^ video analytics as a feasible and interpretable bridge from clinic to home, enabling objective, scalable remote monitoring in ataxia and laying the groundwork for integrated digital endpoints in neurology.

## Author Roles

(1) Research project: A. Conception, B. Organization, C. Execution; (2) Statistical analysis: A. Design, B. Execution, C. Review and critique; (3) Manuscript: A. Writing of the first draft, B. Review and critique.

R.A.: 1A, 1B, 1C, 2A, 2B, 3A

E.F.: 1A, 1B, 1C, 3B

F.P.: 1B, 3B

M.G.‐E.: 1A, 1B, 1C, 2C, 3A, 3B

V.M.: 1C, 3B

J.F.: 1C, 3B

A.T.A.: 1C, 3B

K.H.: 1C, 3B

M.S.: 1C, 3B

L.H.G.: 1C, 3B

D.T.: 1C, 3B

A.T.: 1C, 3B

P.G.: 1C, 3B

H.G.‐M.: 1C, 3B

M.M.S.: 1C, 3B

J.H.‐S.: 1C, 3B

E.H.D.: 1A, 1B, 2C, 3B

T.K.: 1A, 1B, 2C, 3B

## Disclosures

**Ethical Compliance Statement:** This study was approved by the local ethics committees of all participating sites and conducted in accordance with the Declaration of Helsinki. All patients provided written informed consent prior to participation. We confirm that we have read the journal's position on issues involved in ethical publication and affirm that this work is consistent with those guidelines.

**Financial Disclosures and Conflicts of Interest:** Rabia Aziza, Elisa Ferrer, Fereshteh Poursaeed, and Elin H. Davies are employees of Aparito and shareholders of Eli Lilly. Jennifer Faber receives research support from the National Ataxia Foundation (NAF), the Advanced Clinician Scientist Programme (ACCENT, funding code 01EO2107; the ACCENT Program is funded by the German Federal Ministry of Education and Research [BMBF]), and is a fellow of the Hertie Network of Excellence in Clinical Neuroscience. She received consultancy fees from Vico Therapeutics and Biogen, unrelated to the present study. Matthis Synofzik received consulting honoraria from UCB Pharmaceuticals, Prevail Pharmaceuticals, Ionis Pharmaceuticals, Orphazyme Pharmaceuticals, Servier Pharmaceuticals, Reata Pharmaceuticals, AviadoBio, GenOrph, Biohaven, Zevra, Lilly, and Solaxa, all unrelated to the present study. He and Lisa H. Graf are supported by the European Union, project European Rare Disease Research Alliance (ERDERA), GA no. 101156595, funded under call HORIZON‐HLTH‐2023‐DISEASE‐07 (to Matthis Synofzik and Lisa H. Graf). Dagmar Timmann received funding from the DFG, EU, MERCUR, Once Upon a Time Foundation (Raynor Cerebellum Project), and Bernd Fink Foundation unrelated to this work. Andreas Thieme held a clinician scientist position that was supported by the DFG in the framework of the DFG Clinician Scientist Programme UMEA (FU 356/12‐1 and 12‐2) and received travel support from the German Academic Exchange Service (DAAD). Paola Giunti has received grants and honoraria for advisory board participation from Vico Therapeutics, honoraria for advisory board participation from Triplet Therapeutics, grants and personal fees from Reata Pharmaceutical, and grants from Wave. Paola Giunti and Hector Garcia‐Moreno work at University College London Hospitals/University College London, which receives a proportion of funding from the Department of Health's National Institute for Health Research Biomedical Research Centre's funding scheme. Paola Giunti received funding from CureSCA3 in support of Hector Garcia‐Moreno's work. Magda M. Santana was supported by Fundação para a Ciência e Tecnologia, Portugal (2023.06020.CEECIND/CP2832/CT0010). Thomas Klockgether received research support from the Bundesministerium für Bildung und Forschung. All other authors declare no conflicts of interest. This publication is an outcome of the European Spinocerebellar Ataxia Type 3/Machado‐Joseph‐Disease Initiative (ESMI), an EU Joint Programme—Neurodegenerative Disease Research (JPND) project (see www.jpnd.eu). ESMI is supported through the following funding organizations under the aegis of JPND: Germany, Federal Ministry of Education and Research (BMBF, funding codes 01ED1602A/B); Netherlands, the Netherlands Organization for Health Research and Development; and United Kingdom, Medical Research Council (MR/N028767/1). Marcus Grobe‐Einsler received research support from the German Ministry of Education and Research (BMBF) within the European Joint Program for Rare Diseases (EJP‐RD) 2021 Transnational Call for Rare Disease Research Projects (funding number: 01GM2110), and received consulting honoraria from Biogen, all unrelated to this study. Some of the authors of this article are members of the European Reference Network for Rare Neurological Diseases (ERN‐RND). Author disclosures are available in the Supporting Information.

## Supporting information


**Figure S1.** SARAhome Gait and Stance setup.
**Figure S2.** Atom5TM clinician's portal for SARAhome to quality check and score gait videos.
**Figure S3.** Data cleaning example of a gait video where the pose estimation framework detects the coat hanger in the background as a second person, which is corrected in the data cleaning step.
**Figure S4.** SARAhome gait score distribution. Number of videos collected per clinician‐rated gait score, separated by task type: normal gait (green), tandem gait (orange), and no task (pink). ‘No task’ denotes videos in which the participants indicated they were unable to perform the task. Normal and tandem gait are shown together within each score level for comparison.
**Figure S5.** Endpoint derivation: Gait videos at severities 0, 5, and 7. Base of support (inter‐ankle distance) is computed from OpenPose ankle landmarks, normalized by shoulder width to yield a scale‐independent, dimensionless measure. The horizontal axis represents time; panels differ in length because more severe levels take longer to complete the task. The transient peak in the score‐0 (normal gait) panel coincides with the turn: as the shoulders rotate during turning, the projected inter‐shoulder distance used for normalization shortens, transiently inflating the normalized base‐of‐support value. Such turn segments are detected and excluded prior to feature aggregation.
**Figure S6.** Endpoint derivation: Stance videos at severities 0–3 with the corresponding shoulder sway endpoints across normal, feet together and tandem conditions from the same timepoint of a sample patient.
**Figure S7.** Endpoints for a subject with severe staggering gait (SARAhome gait score = 5). The normalized foot elevation (blue for the left foot and green for the right foot) and base of support (green), both scaled by shoulder width, are plotted over the course of the task; the red band indicates the detected turn segment and the dashed red vertical line marks its estimated center.
**Figure S8.** Features aggregated per SARAhome item score stance task videos (feet apart, feet together and tandem).
**Figure S9.** Test–retest reliability (ICC (1); one‐way random effects, single measurement, absolute agreement) of pose‐derived video‐level features, by subtask, for (a) gait and (b) stance. Points are the sample ICC estimate; whiskers are 95% cluster‐bootstrap confidence intervals (2000 iterations, resampling participants). Shaded bands denote Koo & Li (2016) reliability tiers (poor, moderate, good, excellent). Negative ICC estimates, indicating negligible between‐participant variance, are truncated to 0. Estimates correspond to the values tabulated in Table S2.
**Figure S10.** Within‐subject change in a leading pose‐derived feature across two visits, for the eight participants (all Bonn) with a repeat assessment 1 year after baseline. Each line connects a participant's per‐visit median feature value (Visit 1 → Visit 2). Colored lines mark participants whose median SARAhome item score changed (green = feature moved concordantly with the score change; red = discordantly), annotated with the score change; gray lines mark unchanged scores. Panels show, for each subtask, a leading severity‐associated feature; for normal gait we show base of support, the more reliable of the two comparably correlated top features (ICC 0.88; Table S2). Remaining panels show arm swing amplitude (tandem gait), and leg alignment variability (feet apart, feet together, tandem stance). Follow‐up visits were identified from capture dates, as the metadata follow‐up flag was not populated. Given the small number of score changes (two to three per subtask), the analysis is descriptive.
**Table S1.** Participant‐level feature–severity correlations by subtask. Spearman's *ρ* (with 95% cluster bootstrap CI over participants, 2000 iterations) and Kendall's τ between each video‐level feature and clinician‐rated SARAhome item scores, after aggregating repeated recordings to the median per participant and score level. Sensitivity *ρ* is from a single median value per participant. *n* = participants per subtask.
**Table S2.** Test–retest reliability of pose‐derived video‐level features within the baseline recording window. Intraclass correlation coefficients (ICC (1); one‐way random effects, single measurement, absolute agreement) computed from repeated home recordings collected within 30 days of each participant's first recording, over which true disease severity is assumed stable. 95% confidence intervals were obtained by cluster bootstrap resampling of participants (2000 iterations); lower bounds are truncated at 0. Reliability was interpreted using thresholds^24^ (poor <0.50; moderate 0.50–0.75; good 0.75–0.90; excellent >0.90). ICCs are reported separately per subtask; subtasks were not pooled.
**Table S3.** Within‐subject concordance between change in the leading pose‐derived feature and change in SARAhome item score across two visits.
**Appendix S1.** Data cleaning.
**Appendix S2.** Feature calculation.

**Data S1.** COI_disclosure.

## Data Availability

The data supporting the results of this study are available upon request from the corresponding author and agreement of the ESMI consortium. The analysis pipeline relies on proprietary software owned by Aparito and therefore cannot be released in full; the authors are willing to provide additional methodological details on request to support independent reproduction of the reported endpoints.
