# Supplementary material for: Remote Video Assessment of Ataxia via SARAhome : Correlation of Pose‐Derived Endpoints with Clinician Severity Ratings
Source: Mov Disord Clin Pract. 2026 Oct 16:10.1002/mdc3.70854. Online ahead of print. doi: 10.1002/mdc3.70854 (PMC13675665; doi:10.1002/mdc3.70854)
Supplement: Supplementary file 2 [file MDC3-9999-0-s002.docx]

**Supplementary Materials**

Figure S1. SARA^home^ Gait and Stance setup

| SARA^home^ Gait Item | | SARA^home^ Stance Item | | |
| --- | --- | --- | --- | --- |
| Normal | Tandem | Normal | Feet together | Tandem |
|  |  |  |  |  |

Figure S2. Atom5^TM^ clinician’s portal for SARA^home^ to quality check and score gait videos

Figure S3. Data cleaning example of a gait video where the pose estimation framework detects the coat hanger in the background as a second person, which is corrected in the data cleaning step.

Figure S4. SARA^home^ gait score distribution. Number of videos collected per clinician-rated gait score, separated by task type: normal gait (green), tandem gait (orange), and no task (pink). 'No task' denotes videos in which the participants indicated they were unable to perform the task. Normal and tandem gait are shown together within each score level for comparison.

Figure S5. Endpoint derivation: Gait videos at severities 0, 5, and 7. Base of support (inter-ankle distance) is computed from OpenPose ankle landmarks, normalized by shoulder width to yield a scale-independent, dimensionless measure. The horizontal axis represents time; panels differ in length because more severe levels take longer to complete the task. The transient peak in the score-0 (normal gait) panel coincides with the turn: as the shoulders rotate during turning, the projected inter-shoulder distance used for normalization shortens, transiently inflating the normalized base-of-support value. Such turn segments are detected and excluded prior to feature aggregation.

Figure S6. Endpoint derivation: Stance videos at severities 0-3 with the corresponding shoulder sway endpoints across normal, feet together and tandem conditions from the same timepoint of a sample patient.

Figure S7. Endpoints for a subject with severe staggering gait (SARA^home^ gait score = 5). The normalized foot elevation (blue for the left foot and green for the right foot) and base of support (green), both scaled by shoulder width, are plotted over the course of the task; the red band indicates the detected turn segment and the dashed red vertical line marks its estimated center.

Figure S8. Features aggregated per SARA^home^ item score stance task videos (feet apart, feet together and tandem).

Figure S9. Test–retest reliability (ICC(1,1); one-way random effects, single measurement, absolute agreement) of pose-derived video-level features, by subtask, for (a) gait and (b) stance. Points are the sample ICC estimate; whiskers are 95% cluster-bootstrap confidence intervals (2,000 iterations, resampling participants). Shaded bands denote Koo & Li (2016) reliability tiers (poor, moderate, good, excellent). Negative ICC estimates, indicating negligible between-participant variance, are truncated to 0. Estimates correspond to the values tabulated in Table S2.

**Figure S10.** Within-subject change in a leading pose-derived feature across two visits, for the eight participants (all Bonn) with a repeat assessment 1 year after baseline. Each line connects a participant's per-visit median feature value (Visit 1 → Visit 2). Colored lines mark participants whose median SARA^home^ item score changed (green = feature moved concordantly with the score change; red = discordantly), annotated with the score change; grey lines mark unchanged scores. Panels show, for each subtask, a leading severity-associated feature; for normal gait we show base of support, the more reliable of the two comparably correlated top features (ICC 0.88; Table S2). Remaining panels show arm swing amplitude (tandem gait), and leg alignment variability (feet apart, feet together, tandem stance). Follow-up visits were identified from capture dates, as the metadata follow-up flag was not populated. Given the small number of score changes (two to three per subtask), the analysis is descriptive.

Table S1. Participant-level feature–severity correlations by subtask.

Spearman's ρ (with 95% cluster bootstrap CI over participants, 2,000 iterations) and Kendall's τ between each video-level feature and clinician-rated SARA^home^ item scores, after aggregating repeated recordings to the median per participant and score level. Sensitivity ρ is from a single median value per participant. n = participants per subtask.

| **Feature** | **Spearman ρ [95% CI]** | **Kendall τ** | **Participants n** | **Sensitivity ρ** |
| --- | --- | --- | --- | --- |
| **Gait — normal walking** | | | | |
| Clear. var. | 0.52 [0.37, 0.64] | 0.39 | 58 | 0.54 |
| BoS | 0.51 [0.35, 0.66] | 0.40 | 58 | 0.49 |
| Shoulder sway | 0.43 [0.24, 0.60] | 0.33 | 58 | 0.40 |
| Adj. clearance | -0.35 [-0.52, -0.15] | -0.26 | 58 | -0.35 |
| Arm swing amp. | 0.33 [0.14, 0.52] | 0.25 | 58 | 0.24 |
| Leg align. var. | 0.19 [-0.01, 0.40] | 0.14 | 58 | 0.26 |
| Arm swing asym. | -0.11 [-0.30, 0.09] | -0.09 | 58 | -0.18 |
| Clear. asym. | -0.03 [-0.24, 0.17] | -0.02 | 58 | 0.00 |
| **Gait — tandem walking** | | | | |
| Arm swing amp. | 0.26 [0.00, 0.50] | 0.21 | 48 | 0.21 |
| Arm swing asym. | 0.23 [0.01, 0.44] | 0.19 | 48 | 0.16 |
| Leg align. var. | -0.19 [-0.40, 0.04] | -0.15 | 48 | -0.13 |
| BoS | 0.18 [-0.07, 0.39] | 0.13 | 48 | 0.18 |
| Adj. clearance | -0.14 [-0.34, 0.08] | -0.11 | 48 | -0.13 |
| Clear. asym. | 0.08 [-0.16, 0.34] | 0.06 | 48 | 0.14 |
| Shoulder sway | 0.04 [-0.22, 0.29] | 0.03 | 48 | 0.05 |
| Clear. var. | -0.02 [-0.25, 0.20] | -0.01 | 48 | 0.19 |
| **Gait — all** | | | | |
| BoS | 0.58 [0.45, 0.70] | 0.45 | 58 | 0.62 |
| Adj. clearance | -0.25 [-0.44, -0.04] | -0.18 | 58 | -0.29 |
| Arm swing amp. | 0.24 [0.02, 0.47] | 0.20 | 58 | 0.06 |
| Arm swing asym. | 0.21 [0.03, 0.38] | 0.16 | 58 | 0.10 |
| Clear. asym. | -0.14 [-0.34, 0.07] | -0.10 | 58 | -0.12 |
| Leg align. var. | -0.11 [-0.29, 0.11] | -0.08 | 58 | -0.10 |
| Shoulder sway | 0.10 [-0.13, 0.34] | 0.09 | 58 | -0.05 |
| Clear. var. | 0.10 [-0.09, 0.29] | 0.08 | 58 | 0.10 |
| **Stance — tandem** | | | | |
| AP sway | 0.51 [0.36, 0.65] | 0.39 | 45 | 0.49 |
| Leg align. var. | 0.51 [0.36, 0.65] | 0.39 | 45 | 0.56 |
| Composite sway | 0.48 [0.32, 0.62] | 0.36 | 45 | 0.47 |
| ML sway | 0.26 [0.05, 0.45] | 0.19 | 45 | 0.32 |
| ML sway vel. | 0.17 [-0.05, 0.37] | 0.13 | 45 | 0.21 |
| BoS | 0.03 [-0.15, 0.22] | 0.03 | 45 | 0.06 |
| **Stance — feet apart** | | | | |
| Leg align. var. | 0.25 [0.07, 0.41] | 0.19 | 56 | 0.19 |
| BoS | 0.17 [-0.02, 0.34] | 0.13 | 56 | 0.34 |
| ML sway | 0.14 [-0.03, 0.32] | 0.11 | 56 | 0.05 |
| ML sway vel. | 0.13 [-0.02, 0.29] | 0.10 | 56 | 0.02 |
| Composite sway | 0.11 [-0.09, 0.30] | 0.09 | 56 | 0.03 |
| AP sway | 0.09 [-0.11, 0.28] | 0.07 | 56 | 0.01 |
| **Stance — feet together** | | | | |
| Leg align. var. | 0.37 [0.16, 0.54] | 0.28 | 54 | 0.46 |
| ML sway | 0.35 [0.19, 0.50] | 0.28 | 54 | 0.27 |
| ML sway vel. | 0.29 [0.09, 0.46] | 0.22 | 54 | 0.26 |
| Composite sway | 0.18 [-0.03, 0.36] | 0.14 | 54 | 0.11 |
| AP sway | 0.16 [-0.06, 0.34] | 0.12 | 54 | 0.08 |
| BoS | 0.14 [-0.06, 0.34] | 0.11 | 54 | 0.29 |
| **Stance — all** | | | | |
| Leg align. var. | 0.36 [0.16, 0.55] | 0.28 | 56 | 0.36 |
| ML sway | 0.22 [0.05, 0.39] | 0.17 | 56 | 0.16 |
| ML sway vel. | 0.21 [0.02, 0.39] | 0.16 | 56 | 0.11 |
| AP sway | 0.19 [0.00, 0.38] | 0.15 | 56 | 0.06 |
| Composite sway | 0.19 [-0.01, 0.37] | 0.14 | 56 | 0.08 |
| BoS | 0.03 [-0.15, 0.21] | 0.02 | 56 | 0.13 |

Abbreviations: BoS, median normalized base of support; AP, anteroposterior; ML, mediolateral; var., variability; asym., asymmetry; amp., amplitude; vel., velocity; adj., adjusted; CI, confidence interval.

Table S2. Test–retest reliability of pose-derived video-level features within the baseline recording window. Intraclass correlation coefficients (ICC(1,1); one-way random effects, single measurement, absolute agreement) computed from repeated home recordings collected within 30 days of each participant’s first recording, over which true disease severity is assumed stable. 95% confidence intervals were obtained by cluster bootstrap resampling of participants (2,000 iterations); lower bounds are truncated at 0. Reliability was interpreted using thresholds^24^ (poor <0.50; moderate 0.50–0.75; good 0.75–0.90; excellent >0.90). ICCs are reported separately per subtask; subtasks were not pooled.

| **Feature** | **Participants, n** | **Recordings, n** | **ICC(1,1) (95% CI)** | **Reliability** |
| --- | --- | --- | --- | --- |
| **Gait — normal walking (turns excluded)** | | | | |
| Adjusted clearance (worse limb) | 58 | 980 | 0.74 (0.63–0.80) | Moderate |
| Clearance asymmetry | 58 | 975 | 0.16 (0.07–0.27) | Poor |
| Clearance variability | 58 | 980 | 0.35 (0.23–0.43) | Poor |
| Arm swing amplitude | 58 | 977 | 0.33 (0.09–0.57) | Poor |
| Arm swing asymmetry | 58 | 961 | 0.11 (0.04–0.16) | Poor |
| Shoulder sway (RMS) | 58 | 982 | 0.15 (0.05–0.44) | Poor |
| Median normalized base of support | 58 | 981 | 0.88 (0.72–0.93) | Good |
| Leg alignment variability | 58 | 982 | 0.30 (0.20–0.39) | Poor |
| **Gait — tandem walking** | | | | |
| Adjusted clearance (worse limb) | 48 | 784 | 0.60 (0.42–0.73) | Moderate |
| Clearance asymmetry | 48 | 784 | 0.08 (0.02–0.14) | Poor |
| Clearance variability | 48 | 784 | -0.02 (0.00–0.35) | Poor |
| Arm swing amplitude | 48 | 783 | 0.51 (0.19–0.68) | Moderate |
| Arm swing asymmetry | 48 | 780 | 0.29 (0.15–0.44) | Poor |
| Shoulder sway (RMS) | 48 | 784 | 0.16 (0.05–0.32) | Poor |
| Median normalized base of support | 48 | 784 | 0.71 (0.42–0.82) | Moderate |
| Leg alignment variability | 48 | 784 | 0.00 (0.00–0.48) | Poor |
| **Stance — feet apart** | | | | |
| Mediolateral sway (RMS) | 56 | 949 | 0.09 (0.00–0.15) | Poor |
| Anteroposterior sway (RMS) | 56 | 949 | 0.35 (0.21–0.47) | Poor |
| Composite sway | 56 | 949 | 0.18 (0.09–0.37) | Poor |
| Mediolateral sway velocity | 56 | 949 | 0.08 (0.06–0.31) | Poor |
| Median normalized base of support | 56 | 949 | 0.54 (0.40–0.64) | Moderate |
| Leg alignment variability | 56 | 949 | 0.25 (0.17–0.32) | Poor |
| **Stance — feet together** | | | | |
| Mediolateral sway (RMS) | 54 | 907 | 0.36 (0.00–0.43) | Poor |
| Anteroposterior sway (RMS) | 54 | 907 | 0.38 (0.22–0.52) | Poor |
| Composite sway | 54 | 907 | 0.35 (0.19–0.45) | Poor |
| Mediolateral sway velocity | 54 | 907 | 0.38 (0.01–0.46) | Poor |
| Median normalized base of support | 54 | 907 | 0.29 (0.15–0.46) | Poor |
| Leg alignment variability | 54 | 907 | 0.24 (0.11–0.39) | Poor |
| **Stance — tandem** | | | | |
| Mediolateral sway (RMS) | 45 | 773 | 0.21 (0.00–0.33) | Poor |
| Anteroposterior sway (RMS) | 45 | 773 | 0.31 (0.18–0.42) | Poor |
| Composite sway | 45 | 773 | 0.23 (0.07–0.33) | Poor |
| Mediolateral sway velocity | 45 | 773 | 0.13 (0.00–0.28) | Poor |
| Median normalized base of support | 45 | 773 | 0.53 (0.39–0.65) | Moderate |
| Leg alignment variability | 45 | 773 | 0.31 (0.17–0.41) | Poor |

Abbreviations: ICC, intraclass correlation coefficient; CI, confidence interval; RMS, root mean square.

Table S3. Within-subject concordance between change in the leading pose-derived feature and change in SARAhome item score across two visits.

| **Subtask** | **Leading feature** | **Participants (n)** | **Score changed (n)** | **Direction of score change** | **Feature concordant (n)** |
| --- | --- | --- | --- | --- | --- |
| Gait, normal | Base of support | 8 | 3 | All worsening | 1 |
| Gait, tandem | Arm swing amplitude | 7 | 2 | All worsening | 0 |
| Stance, feet apart | Leg alignment variability | 8 | 2 | All worsening | 1 |
| Stance, feet together | Leg alignment variability | 8 | 2 | All worsening | 2 |
| Stance, tandem | Leg alignment variability | 8 | 2 | All worsening | 1 |

Eight participants (all from the Bonn site) contributed a repeat assessment approximately one year after baseline; follow-up visits were identified from capture dates. “Feature concordant” counts participants whose leading-feature median moved in the same direction as their SARAhome item-score change. Given the small number of score changes, the analysis is descriptive.

Appendix 1. Data cleaning

Rigorous data preparation was essential to ensure the integrity and usability of the SARA^home^ video dataset for both manual and automated analyses.

- **Manual Labelling & Annotation:** Each video was manually labelled to capture key metadata, including task start and end times, use of assistive devices, and occurrence of falls. This step ensured that only relevant segments were included for analysis.
- **Semi-Automated Corrections:** Videos were reviewed to identify and trim non-relevant footage, such as delayed task initiation or interruptions. Criteria for exclusion included minimum task duration and absence of task performance. When multiple individuals appeared in frame, the correct participant was identified and tracked throughout the video.
- **Pose Estimation & Quality Control:** Pose estimation algorithms were applied to detect and track body landmarks. Outputs included annotated frames and plots of landmark coordinates over time, which were manually reviewed to verify consistent tracking of the correct individual. For example, heel landmark trajectories were inspected to confirm accurate tracking during gait tasks.
- **Error Handling & Data Cleaning:** Common errors, such as misidentification of objects (e.g., coat hangers, paintings) as people, or tracking the wrong individual in multi-person frames, were flagged and corrected. Corrections were logged to maintain a transparent audit trail.

Appendix 2. Feature calculation
            1. Gait Features

Gait features were computed from normal and tandem walking tasks. For normal walking, turn segments were detected and excluded prior to feature aggregation. All features below represent single video-level summary values.

**Adjusted Clearance of the Worse Limb**

Normalized foot clearance of the limb with the lower median hip-to-ankle distance during the gait cycle. A shorter hip-to-ankle distance indicates reduced leg extension during swing phase, reflecting the more impaired limb. Lower values indicate greater impairment.

***Equation:*** $\text{adj}\text{\_}\text{clearance}\text{\_}\text{worse}=\frac{\text{median}\left( d_{\text{hip-ankle, worse}} \right)}{\text{shoulder}\text{\_}\text{width}}$

*where:*

$d_{\text{hip-ankle, worse}}$= frame-wise hip-to-ankle Euclidean distance of the limb with the lower median value across the task

*shoulder_width* = frame-wise Euclidean distance between left and right shoulder landmarks

**Clearance Asymmetry**

Normalized absolute difference in median foot clearance between the left and right limbs. Values near zero indicate symmetric clearance; higher values indicate asymmetric limb performance.

***Equation:*** $\text{clearance}\text{\_}\text{asym}=\frac{\left| \text{median}\left( d_{\text{R}} \right)-\text{median}\left( d_{\text{L}} \right) \right|}{\text{median}\left( d_{\text{R}} \right)+\text{median}\left( d_{\text{L}} \right)}$

*where:*

$d_{R},d_{L}$ = frame-wise hip-to-ankle distance for the right and left limbs, respectively

**Clearance Variability**

Standard deviation of foot clearance computed over the pooled (concatenated) left and right limb time series. Higher variability indicates more irregular foot elevation across steps, reflecting gait instability.

***Equation:*** $\text{clearance}\text{\_}\text{var}=\text{SD}\left( d_{\text{R}}\cup d_{\text{L}} \right)$

*where:*

$d_{R}\cup d_{L}$ = concatenated hip-to-ankle distance time series from both limbs

**Arm Swing Amplitude**

Mean range of the arm swing angle across both limbs. The arm angle is defined as the angle at the elbow formed by the wrist, elbow, and shoulder landmarks. Lower values indicate reduced arm swing.

***Equation:*** $\text{arm}\text{\_}\text{swing}\text{\_}\text{amp}=\frac{\text{range}\left( \theta_{\text{arm,R}} \right)+\text{range}\left( \theta_{\text{arm,L}} \right)}{2}$

*where:*

$\theta_{arm,R}$ *and* $\theta_{arm,L}$ = frame-wise arm angle (wrist–elbow–shoulder) for the right and left arms

*range(∙)* = maximum minus minimum value across the task

**Arm Swing Asymmetry**

Normalized absolute difference in arm swing range between left and right arms. Values near zero indicate symmetric arm swing; higher values indicate lateralized impairment.

***Equation:*** $\text{arm}\text{\_}\text{swing}\text{\_}\text{asym}=\frac{\left| \text{range}\left( \theta_{\text{arm,R}} \right)-\text{range}\left( \theta_{\text{arm,L}} \right) \right|}{\text{range}\left( \theta_{\text{arm,R}} \right)+\text{range}\left( \theta_{\text{arm,L}} \right)}$

**Shoulder Sway (RMS)**

Root mean square of the horizontal shoulder sway angle, measured as the angle formed by the left and right shoulder landmarks relative to the horizontal. Captures mediolateral trunk oscillation during gait; higher values indicate greater postural instability.

***Equation:*** $\text{shoulder}\text{\_}\text{sway}\text{\_}\text{rms}=\sqrt{\frac{1}{N}\sum_{i=1}^{N} \theta_{\text{shoulder},i}^{2}}$

*where:*

$\theta_{shoulder,i}$ = shoulder tilt angle at frame i, computed as the angle of the line connecting LEFT_SHOULDER and RIGHT_SHOULDER relative to the horizontal

*N* = total number of frames (excluding turns for normal gait)

**Median Normalized Base of Support**

Median ankle-to-ankle distance normalized by shoulder width. Wider base of support indicates compensatory strategies for balance impairment.

***Equation:*** $nbos_{median}=median\left( \frac{\left\| p_{L,ankle}-p_{R,ankle} \right\|}{shoulder_{w}idth} \right)$

*where:*

$p_{L\_ANKLE},p_{R\_ANKLE}$ = 2D coordinates of the left and right ankle landmarks

**Leg Alignment Variability**

Standard deviation of the knee alignment angle across the task. The knee alignment angle is defined as the angle at the knee formed by the ankle, knee, and hip landmarks. Higher variability reflects irregular knee control during gait.

***Equation:*** $\text{leg}\text{\_}\text{align}\text{\_}\text{var}=\text{SD}\left( \angle\left( \text{ANKLE},\text{KNEE},\text{HIP} \right) \right)$

*where:*

*∠(ANKLE, KNEE, HIP)* = frame-wise angle at the knee vertex, computed bilaterally and pooled

2. Stance Features

Stance features were computed from three stance conditions: feet apart (normal stance), feet together, and tandem stance. All features represent single video-level summary values computed over the full duration of each stance task.

**Mediolateral Sway (RMS)**

Root mean square of the mediolateral sway angle, estimated from the tilt of the shoulder line (left-to-right shoulder) relative to the horizontal. Captures lateral trunk oscillation during stance.

***Equation:*** $\text{ml}\text{\_}\text{sway}\text{\_}\text{rms}=\sqrt{\frac{1}{N}\sum_{i=1}^{N} \theta_{\text{ML},i}^{2}}$

*where:*

$\theta_{ML,i}$ = mediolateral shoulder tilt angle at frame i

**Anteroposterior Sway (RMS)**

Root mean square of the anteroposterior sway angle, estimated from the angle between the neck and mid-hip landmarks. Captures forward–backward trunk oscillation during stance.

***Equation:*** $\text{ap}\text{\_}\text{sway}\text{\_}\text{rms}=\sqrt{\frac{1}{N}\sum_{i=1}^{N} \theta_{\text{AP},i}^{2}}$

*where:*

$\theta_{AP,i}$ = anteroposterior trunk angle at frame i, computed as the angle of the line connecting NECK and MID_HIP

**Composite Sway**

Combined sway metric computed as the Euclidean norm of the mediolateral and anteroposterior RMS sway values. Provides a single summary of overall postural instability.

***Equation:*** $\text{composite}\text{\_}\text{sway}=\sqrt{{\text{ml}\text{\_}\text{sway}\text{\_}\text{rms}}^{2}+{\text{ap}\text{\_}\text{sway}\text{\_}\text{rms}}^{2}}$

**Mediolateral Sway Velocity**

Mean angular velocity of mediolateral sway, computed as the average absolute frame-to-frame change in the shoulder tilt angle divided by the inter-frame time interval. Higher values indicate more rapid lateral postural corrections.

***Equation:*** $\text{sway}\text{\_}\text{velocity}\text{\_}\text{ml}=\frac{1}{N-1}\sum_{i=1}^{N-1} \frac{\left| \theta_{\text{ML},i+1}-\theta_{\text{ML},i} \right|}{\Delta t}$

*where:*

*Δt* = time interval between consecutive frames (1/frame rate)

**Median Normalized Base of Support (Stance)**

Median ankle-to-ankle distance normalized by shoulder width during stance. Interpretation is analogous to the gait feature but applied to static balance tasks.

***Equation:*** $nbos_{median}=median\left( \frac{\left\| p_{L,ankle}-p_{R,ankle} \right\|}{shoulder_{w}idth} \right)$

**Leg Alignment Variability (Stance)**

Standard deviation of the knee alignment angle during stance. Interpretation is analogous to the gait feature but applied to static balance tasks, where increased variability reflects compensatory postural adjustments.

***Equation:*** $\text{leg}\text{\_}\text{align}\text{\_}\text{var}=\text{SD}\left( \angle\left( \text{ANKLE},\text{KNEE},\text{HIP} \right) \right)$
